# Ki-67 and breast cancer prognosis: does it matter if Ki-67 level is examined using preoperative biopsy or postoperative specimen?

**DOI:** 10.1007/s10549-022-06519-1

**Published:** 2022-01-13

**Authors:** Soon Bo Choi, Jung Min Park, Jee Hyun Ahn, Jieon Go, Jeeye Kim, Hyung Seok Park, Seung Il Kim, Byeong-Woo Park, Seho Park

**Affiliations:** Division of Breast Surgery, Department of Surgery, Yonsei University College of Medicine, Seoul, Republic of Korea

**Keywords:** Ki-67, Breast cancer, Prognosis, Biopsy, Surgical specimen

## Abstract

**Purpose:**

This study aimed to identify the association between Ki-67 level and the prognosis of patients with breast cancer, regardless of the timing of Ki-67 testing (using preoperative biopsy vs. postoperative specimen).

**Methods:**

A total of 4177 patients underwent surgery between January 2008 and December 2016. Immunohistochemical Ki-67 levels, using either preoperative (1673) or postoperative (2831) specimens, were divided into four groups using cutoff points of 10%, 15%, and 20%.

**Results:**

Groups with higher-Ki-67 levels, in both the pre- and postoperative periods, showed significantly larger tumor size, higher grade, more frequent hormone receptor-negativity and human epidermal growth factor receptor 2 overexpression, and active adjuvant treatments than groups with lower-Ki-67 levels. High-Ki-67 levels were also significantly associated with poor survival, irrespective of the timing of specimen examination.

**Conclusion:**

Despite the problems associated with Ki-67, Ki-67 level is an important independent prognostic factor, regardless of the timing of Ki-67 testing, i.e., preoperative or postoperative testing.

## Introduction

Breast cancer is a heterogeneous disease [1–3]. The nature of breast cancer varies not only between different histologic types or subtypes but also varies from person-to-person. Presently, the treatment of breast cancer requires a multifaceted approach and early detection of breast cancer is crucial [4, 5]. Gene profiling and immunohistochemical (IHC) studies are conducted to investigate various biologic characteristics and predict the prognoses of breast cancer patients [4]. At present, four breast cancer subtypes have been identified, namely, luminal A, luminal B, human epidermal growth factor receptor 2 (HER2) enriched, and basal-like. Accordingly, various treatment strategies have been implemented and the prognosis has been predicted [4–6].

Among various characteristics of breast cancer, progression and aggression are currently evaluated using the biologic indicator Ki-67 to determine the degree of tumor proliferation [7]. Ki-67 is a non-histone nuclear cortex protein, which is encoded by the *MKI67* gene and is expressed in the cell nucleus during the G1, S, G2, and M phases of the cell cycle, but not in the G0 phase [8, 9].

Since 2007, many studies have been conducted to determine the cutoff value of Ki-67 that can be used as a prognostic factor [10–13]. Based on these studies, in the 2011 St. Gallen conference, Ki-67 level became the standard factor for differentiating between luminal B and luminal A types in patients with estrogen receptor-positive (ER +) and HER2-negative (HER2-) breast cancer; the recommended cutoff value for this classification was 14% [6]. However, the accuracy, analytical validity, and reproducibility of the methods for detecting Ki-67 remained controversial [9]. Therefore, for determining its analytical validity, the international Ki-67 in the Breast Cancer Working Group has provided guidelines for Ki-67 scoring [14]. Nevertheless, challenges with global standardization still persist and the 2015 St. Gallen conference recommended that a lab-specific median Ki-67 cutoff value should be determined through standardization procedures in each institution [15].

Keeping the analytical problems aside, the timing of the test also remains questionable, making its reproducibility debatable [9]. Although there have been previous studies on the concordance of preoperative and postoperative Ki-67 levels of only the same patient, it remains questionable whether the analysis of Ki-67 level performed at one time can represent the entire proliferation index of breast cancer [16–19]. In addition, several studies have tackled the timing of only the test pathologically; however, information on clinical approaches remains scarce [17–19].

The purpose of this study was to resolve the question on the suitable timing of Ki-67 testing and to evaluate whether the Ki-67 index is an appropriate proliferation index of breast cancer, i.e., a factor influencing the prognosis of breast cancer, regardless of the time of testing.

### Methods

#### Patients

We retrospectively reviewed the records of 4177 patients with pathologically confirmed invasive breast cancer who underwent surgery at the Department of Surgery, Yonsei University College of Medicine, between January 2008 and December 2016. Patients whose Ki-67 levels were investigated for breast carcinoma were included, regardless of when the Ki-67 levels were examined. We excluded patients for whom Ki-67 was not assessed, those who underwent neoadjuvant chemotherapy, and patients with in situ carcinoma, microinvasive carcinoma, recurrent, or metastatic disease. The study population comprised patients with stage I–IIIc breast cancer according to the American Joint Committee on Cancer, 8th edition, which is a commonly used pathological staging system.

The time of measuring Ki-67 levels was divided into pre- and postoperative periods. Preoperative Ki-67 (Pre-Ki-67) was defined as Ki-67 levels measured in tissues obtained through core needle biopsy (CNB), vacuum-assisted biopsy (VAB), or rarely excisional/incisional biopsy. Further, postoperative Ki-67 (Post-Ki-67) was defined as Ki-67 levels measured in tissue specimens obtained from surgery. IHC analysis was performed on biopsy and permanent specimen. Ki-67 levels were arbitrarily stratified and patients were divided into four subgroups according to Ki-67 level: < 10% (lowest), 10– < 15% (medium–low), 15– < 20% (medium–high), and ≥ 20% (highest).

This study was approved by the Institutional Review Board of Severance Hospital, Seoul, Republic of Korea (IRB No. 4-2020-1293). The requirement for informed consent was waived due to the retrospective nature of the study.

#### Clinicopathological characteristics

Patient demographics and clinical information including treatment modalities and expression of ER, progesterone receptor (PR), HER2, and Ki-67 levels were obtained from electronic medical records and pathology reports. Positive ER and PR were defined as IHC nuclear-stained cells ≥ 1%, based on the American Society of Clinical Oncology/College of American Pathologists (ASCO/CAP) guidelines [20]. According to the ASCO/CAP guidelines, HER2 was scored as 0, 1 +, 2 +, or 3 + [21]. In patients with an HER2 2 + result, fluorescence in situ hybridization (FISH) or silver in situ hybridization (SISH) was performed. HER2 gene amplification was defined as a HER2 gene/chromosome 17 copy number ratio ≥ 2.0 or a HER2 gene/chromosome 17 copy number ratio < 2.0, but with average HER2 copy number ≥ 6.0 signals/cell according to the ASCO/CAP guidelines [21]. HER2 was considered positive in cases with an IHC score of 3 + or gene amplification detected by FISH or SISH.

IHC was performed for measuring Ki-67 levels in both preoperative biopsy tissue and postsurgical specimens using a primary MIB-1 antibody (Dako Denmark A/S, Glostrup, Denmark), following protocols established by the Department of Pathology at our institution. Using a visual grading system, a specialized pathologist highly experienced in breast pathology determined the Ki-67 levels by counting the number of positively stained nuclei on hotspot and expressed it as a percentage of total tumor cells in the specimen (Ki-67 level; %).

#### Endpoints

The primary endpoint was disease-free survival (DFS) and overall survival (OS) in relation to the four subgroups of Pre-Ki-67 and Post-Ki-67 levels. Recurrence was divided into loco-regional recurrence and distant metastasis. Contralateral breast cancer was not included as a recurrence. The follow-up period was set from the day of surgery to the date of the last hospital visit, regardless of the department visited. It was possible to confirm the exact patient’s event by following up regardless of the department. DFS was defined as the period from the date of surgery to the date of the first observation of recurrence, death, or the last follow-up date without evidence of any events, while OS was defined as the period from the date of surgery to the date of death or the last follow-up.

#### Statistical analyses

The clinicopathological information, including demographic, clinical, and treatment-related data of the patients, were analyzed based on the four subgroups for Pre-Ki-67 and Post-Ki-67 levels. The variables were compared using a Chi-squared test, *t* test, or analysis of variance. The Kaplan–Meier method was used to predict survival rates, and a log-rank test was used to compare the four subgroups. In a multivariate analysis, Cox proportional hazard regression was used for adjustment of other factors. Cox regression analysis was used to calculate hazard ratios and 95% confidence intervals. Values were two-sided and statistical significance was defined at *P* < 0.05. All statistical analyses were performed using SPSS software, version 25 (IBM Corp., Armonk, NY, USA).

## Results

### Characteristics of patients

The clinicopathological characteristics of patients are summarized in Table 1. There were 1346 (32.2%) patients who underwent Ki-67 testing in the preoperative period alone, 2504 (59.9%) patients who underwent Ki-67 testing in the postoperative period alone, and 327 (7.9%) patients who underwent Ki-67 testing both in the preoperative and postoperative periods. Finally, a total of 1673 and 2831 patients were included in the Pre-Ki-67 and Post-Ki-67 groups, respectively. The median Ki-67 of the Pre-Ki-67 group was 10.00 and that of the Post-Ki-67 group was 12.55. The mean ± standard deviation (SD) of the age of the patients at diagnosis was 52.3 ± 11.1 years and the mean ± SD of body mass index (BMI) was 23.4 ± 3.4 kg/m^2^.

**Table 1:** Clinicopathological characteristics of the patients stratified by the four subgroups of preoperative and postoperative Ki-67 levels

Factors	Pre-Ki-67 (*n* = 1,673)					Post-Ki-67 (*n* = 2,831)				
	< 10%	10%– < 15%	15%– < 20%	≥ 20%	*P* value	< 10%	10%– < 15%	15%– < 20%	≥ 20%	*P* value
	(*n* = 770)	(*n* = 256)	(*n* = 118)	(*n* = 529)		(*n* = 995)	(*n* = 467)	(*n* = 198)	(*n* = 1171)	
Age (years)										
< 50	348 (45.2)	130 (50.8)	56 (47.5)	241 (45.6)	0.453	404 (40.6)	217 (46.5)	88 (44.4)	553 (47.2)	0.016
≥ 50	422 (54.8)	126 (49.2)	62 (52.5)	288 (54.4)		591 (59.4)	250 (53.5)	110 (55.6)	618 (52.8)	
BMI (kg/m^2^)										
Normal (< 23)	415 (53.9)	141 (55.1)	60 (50.8)	292 (55.2)	0.837	497 (49.9)	227 (48.6)	102 (51.5)	601 (51.3)	0.758
Obesity (≥ 23)	355 (46.1)	115 (44.9)	58 (49.2)	237 (44.8)		498 (50.1)	240 (51.4)	96 (48.5)	570 (48.7)	
Tumor size (cm)										
≤ 2	629 (81.7)	201 (78.5)	81 (68.6)	342 (64.7)	< 0.001	854 (85.8)	346 (74.1)	144 (72.7)	765 (65.3)	< 0.001
> 2	141 (18.3)	55 (21.5)	37 (31.4)	187 (35.3)		141 (14.2)	121 (25.9)	54 (27.3)	406 (34.7)	
Lymph node status										
Negative	613 (79.6)	188 (73.4)	90 (76.3)	425 (80.3)	0.118	797 (80.1)	358 (76.7)	151 (76.3)	891 (76.1)	0.135
Positive	157 (20.4)	68 (26.6)	28 (23.7)	104 (19.7)		198 (19.9)	109 (23.3)	47 (23.7)	280 (23.9)	
Histologic grade										
Grades 1 & 2	728 (94.5)	221 (86.3)	87 (73.7)	245 (46.3)	< 0.001	971 (97.6)	430 (92.1)	167 (84.3)	583 (49.8)	< 0.001
Grade 3	42 (5.5)	35 (13.7)	31 (26.3)	284 (53.7)		24 (2.4)	37 (7.9)	31 (15.7)	588 (50.2)	
ER or PR status										
Negative	44 (5.7)	24 (9.4)	26 (22.0)	242 (45.7)	< 0.001	48 (4.8)	46 (9.9)	24 (12.1)	495 (42.3)	< 0.001
Positive	726 (94.3)	232 (90.6)	92 (78.0)	287 (54.3)		947 (95.2)	421 (90.1)	174 (87.9)	676 (57.7)	
HER2										
Negative	724 (94.0)	228 (89.1)	79 (66.9)	394 (74.5)	< 0.001	951 (95.6)	414 (88.7)	166 (83.8)	861 (73.5)	< 0.001
Positive	46 (6.0)	28 (10.9)	39 (33.1)	135 (25.5)		44 (4.4)	53 (11.3)	32 (16.2)	310 (26.5)	
Operation status										
BCS	500 (64.9)	148 (57.8)	69 (58.5)	338 (63.9)	0.144	600 (60.3)	259 (55.5)	116 (58.6)	669 (57.1)	0.285
TM	270 (35.1)	108 (42.2)	49 (41.5)	191 (36.1)		395 (39.7)	208 (44.5)	82 (41.4)	502 (42.9)	
Radiation therapy										
Not done	212 (27.5)	83 (32.4)	38 (32.2)	153 (28.9)	0.416	344 (34.6)	168 (36.0)	80 (40.4)	430 (36.7)	0.424
Done	558 (72.5)	173 (67.6)	80 (67.8)	376 (71.1)		651 (65.4)	299 (64.0)	118 (59.6)	741 (63.3)	
Endocrine therapy										
Not done	55 (7.1)	24 (9.4)	25 (21.2)	241 (45.6)	< 0.001	56 (5.6)	47 (10.1)	26 (13.1)	498 (42.5)	< 0.001
Done	715 (92.9)	232 (90.6)	93 (78.8)	288 (54.4)		939 (94.4)	420 (89.9)	172 (86.9)	673 (57.5)	
Chemotherapy										
Not done	427 (55.5)	109 (42.6)	24 (20.3)	85 (16.1)	< 0.001	635 (63.8)	234 (50.1)	90 (45.5)	239 (20.4)	< 0.001
Done	343 (44.5)	147 (57.4)	94 (79.7)	444 (83.9)		360 (36.2)	233 (49.9)	108 (54.5)	932 (79.6)	
Biologic therapy										
Not done	734 (95.3)	230 (89.8)	83 (70.3)	413 (78.1)	< 0.001	965 (97.0)	437 (93.6)	170 (85.9)	930 (79.4)	< 0.001
Done	36 (4.7)	26 (10.2)	35 (29.7)	116 (21.9)		30 (3.0)	30 (6.4)	28 (14.1)	241 (20.6)	

*BMI* body mass index, *ER* estrogen receptor, *PR* progesterone receptor, *HER2* human epidermal growth factor receptor type 2, *BCS* breast-conserving surgery, *TM* total mastectomy

In the Pre-Ki-67 group, the lowest, medium–low, medium–high, and highest Ki-67 levels were found in 770 (46.0%), 256 (15.3%), 118 (7.1%), and 529 patients (31.6%), respectively, and in the Post-Ki-67 group, the lowest, medium–low, medium–high, and highest Ki-67 levels were found in 995 (35.1%), 467 (16.5%), 198 (7.0%), and 1171 patients (41.4%), respectively. The means ± SDs of age in the Pre-Ki-67 and Post-Ki-67 groups were 52.2 ± 10.6 years and 52.6 ± 11.3 years, respectively, while the means ± SDs of BMI in each of the two groups were 23.3 ± 3.4 kg/m^2^ and 23.4 ± 3.4 kg/m^2^, respectively. Age and BMI did not significantly differ with Ki-67 levels in both the Pre-Ki-67 and Post-Ki-67 groups.

Regarding pathological parameters, most of the factors showed statistically significant differences according to the Ki-67 levels and similar trends were observed in both groups. Tumors sized ≤ 2 cm, histologic grade I/II, hormone receptor-positivity, and HER2-negativity were significantly associated with lower Ki-67 levels. In contrast, tumors sized > 2 cm, histologic grade III, hormone receptor-negativity, and overexpression of HER2 were significantly associated with the highest Ki-67 levels. Unlike other pathological factors, lymph node status was not significantly related to Ki-67 levels in any period.

Regarding treatment modalities, both periods showed similar trends. The frequency of local therapy did not significantly differ according to Ki-67 levels. However, the use of systemic therapies such as chemo-endocrine therapy and targeted therapy showed significant differences across Ki-67 levels as expected. More than 90% of the patients, who received endocrine therapy, tended to have the lowest level of Ki-67. In contrast, most patients who received chemotherapy and biologic therapy had medium–high or highest level of Ki-67.

### Survival according to Pre-Ki-67 and Post-Ki-67 levels

**Fig. 1:**
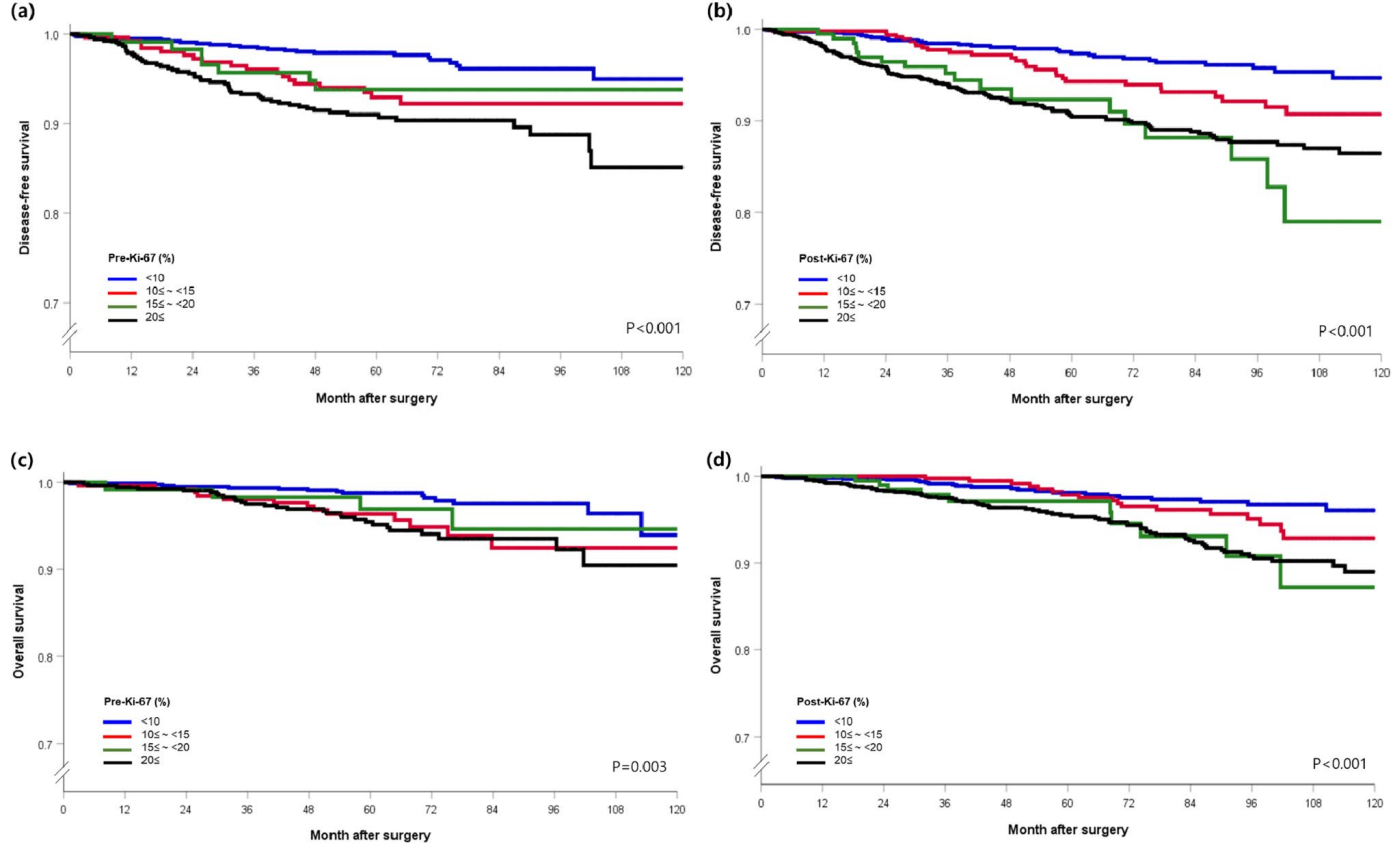
Survival curves according to the four subgroups of Pre-Ki-67 and Post-Ki-67 levels (**a**) Disease-free survival in the Pre-Ki-67 group. (**b**) Disease-free survival in the Post-Ki-67 group. (**c**) Overall survival in the Pre-Ki-67 group. (**d**) Overall survival in the Post-Ki-67 group. Pre-Ki-67, preoperative Ki-67; Post-Ki-67, postoperative Ki-67

Figure 1 shows the 10-year DFS and OS according to Ki-67 levels in both the Pre-Ki-67 and Post-Ki-67 groups*.* The mean follow-up duration of the patients was 69.3 months (SD: 30.4 months). The mean follow-up durations in the Pre-Ki-67 and Post-Ki-67 groups were 70.7 months (SD: 23.1 months) and 69.5 months (SD: 33.5 months), respectively. DFS and OS were significantly different between the groups and similar trends were observed in each period. Although the remaining three Pre-Ki-67 groups had a worse prognosis compared with the lowest Ki-67 group, there was no statistical significance observed between the lowest and medium–low groups (Fig. 1a and c). Likewise, with regard to DFS and OS of the Post-Ki-67 group, it was confirmed that the three remaining groups all had a worse prognosis compared with that of the lowest group (Fig. 1b and d). However, there was no significant difference between the lowest and low–medium groups in the OS of the Post-Ki-67 group (Fig. 1d).

### Multivariate and subgroup analyses

The results of multivariate analyses for DFS and OS are presented in Tables 2 and 3, respectively, which were similar in all the Pre-Ki-67 and Post-Ki-67 subgroups. When adjusting for clinicopathological prognostic factors, a high-Ki-67 level was determined to be an independent prognostic factor for both DFS and OS. However, the degree of hazard ratio was not proportional to Ki-67 levels. Larger tumor size and high nodal stage were also independent and significant prognostic factors for survival. Hormone receptor-positivity demonstrated an increased hazard ratio for DFS and OS without statistical significance. All systemic therapies had decreased hazard ratios for DFS and OS without statistical significance. Age was seen as an important prognostic factor for OS as compared with that for DFS.

**Table 2:** Multivariate analysis for DFS according to Pre-Ki-67 and Post-Ki-67

Factor	Pre-Ki-67			Post-Ki-67		
	Hazard ratio	95% CI	*P* value	Hazard ratio	95% CI	*P* value
Ki-67 (%)						
< 10	1			1		
10– < 15	2.227	1.194–4.154	0.012	1.675	1.002–2.802	0.049
15– < 20	1.830	0.771–4.342	0.171	3.009	1.666–5.433	< 0.001
≥ 20	2.876	1.628–5.081	< 0.001	2.340	1.452–3.693	< 0.001
Age (years)						
< 50 vs. ≥ 50	1.053	0.702–1.579	0.803	1.234	0.918–1.658	0.163
Tumor size (cm)						
≤ 2 vs. > 2	2.398	1.569–3.665	< 0.001	1.586	1.164–2.162	0.003
Lymph node status						
Negative vs. Positive	1.791	1.106–2.901	0.018	1.817	1.284–2.570	0.001
Histologic grade						
Grades 1 and 2 vs. 3	0.904	0.549–1.488	0.690	1.429	0.983–2.078	0.059
ER/PR status						
Negative vs. Positive	1.217	0.261–5.669	0.803	1.109	0.361–3.407	0.952
HER2 status						
Negative vs. Positive	0.755	0.439–1.298	0.310	0.633	0.421–0.952	0.028
Operation status						
BCS vs. TM	1.147	0.595–2.211	0.682	1.443	0.925–2.249	0.106
Radiation therapy						
Not done vs. Done	0.531	0.280–1.005	0.052	0.646	0.421–0.990	0.045
Endocrine therapy						
Not done vs. Done	0.556	0.122–2.538	0.449	0.579	0.192–1.749	0.333
Chemotherapy						
Not done vs. Done	0.958	0.561–1.638	0.877	0.893	0.608–1.313	0.566

*CI* confidence interval, *ER* estrogen receptor, *PR* progesterone receptor, *HER2* human epidermal growth factor receptor type 2, *BCS* breast-conserving surgery, *TM* total mastectomy, *DFS* disease-free survival

**Table 3:** Multivariate analysis for OS according to Pre-Ki-67 and Post-Ki-67

Factor	Pre-Ki-67			Post-Ki-67		
	Hazard ratio	95% CI	*P* value	Hazard ratio	95% CI	*P* value
Ki-67 (%)						
< 10	1			1		
10– < 15	2.640	1.242–5.611	0.012	1.412	0.747–2.670	0.288
15– < 20	1.668	0.539–5.164	0.375	2.382	1.113–5.095	0.025
≥ 20	2.310	1.117–4.781	0.024	2.139	1.236–3.701	0.007
Age (years)						
< 50 vs. ≥ 50	1.739	1.019–2.967	0.042	1.412	0.974–2.048	0.068
Tumor size (cm)						
≤ 2 vs. > 2	3.107	1.782–5.419	< 0.001	1.749	1.187–2.577	0.005
Lymph node status						
Negative vs. Positive	2.362	1.281–4.356	0.006	2.486	1.624–3.806	< 0.001
Histologic grade						
Grades 1 and 2 vs. 3	0.892	0.472–1.683	0.724	1.569	0.990–2.488	0.055
ER/PR status						
Negative vs. Positive	1.474	0.242–8.967	0.674	1.280	0.358–4.576	0.704
HER2 status						
Negative vs. Positive	0.722	0.355–1.469	0.369	0.707	0.428–1.166	0.174
Operation status						
BCS vs. TM	1.194	0.538–2.646	0.663	1.381	0.789–2.416	0.259
Radiation therapy						
Not done vs. Done	0.684	0.315–1.487	0.338	0.576	0.338–0.983	0.043
Endocrine therapy						
Not done vs. Done	0.379	0.064–2.236	0.284	0.489	0.140–1.714	0.264
Chemotherapy						
Not done vs. Done	0.731	0.370–1.447	0.369	0.600	0.374–0.963	0.034

*CI* confidence interval, *ER* estrogen receptor, *PR* progesterone receptor, *HER2* human epidermal growth factor receptor type 2, *BCS* breast-conserving surgery, *TM* total mastectomy, *OS* overall survival

**Fig. 2:**
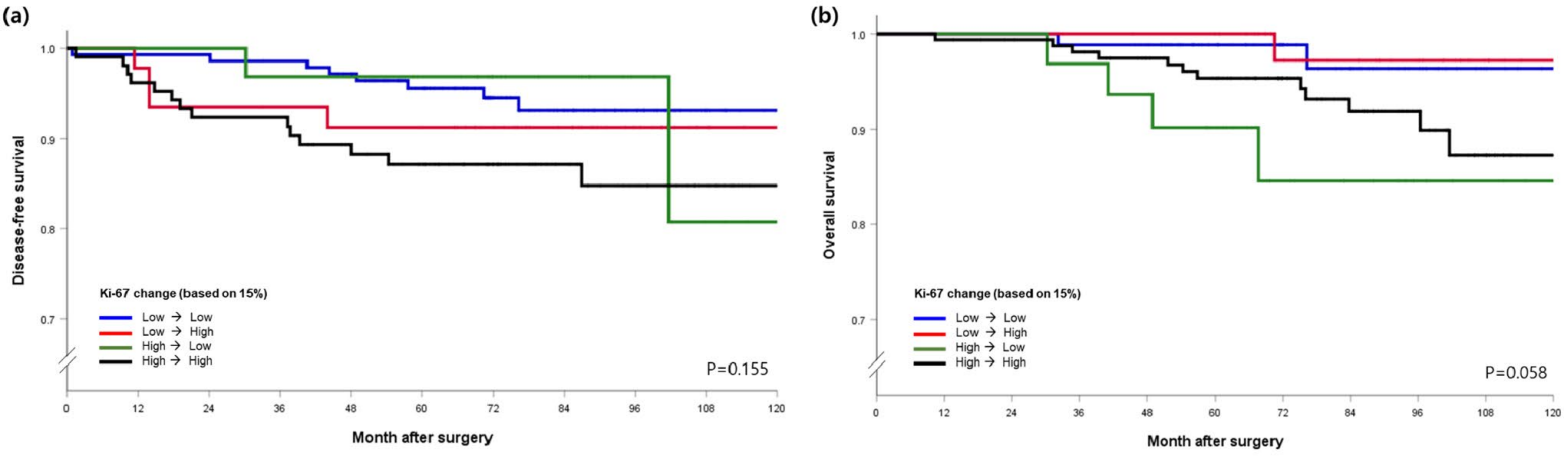
Survival curves according to Ki-67 level change. (**a**) Disease-free survival. (**b**) Overall survival

Although only 327 (7.9%) patients in the entire study population were examined for Ki-67 levels using both preoperative and postoperative specimens, we exploratively analyzed survival outcomes according to combined Pre-Ki-67 and Post-Ki-67 status. Given that patients with medium–low and medium–high levels of Ki-67 showed the worst outcomes, a cutoff point of Ki-67 was considered: < 15% (low) versus ≥ 15% (high). Of 327 cases, 43.7% and 32.4% had low and high-Ki-67 levels, respectively. However, 14.1% and 9.8% of patients had postoperatively increased and decreased Ki-67 levels, respectively. Figure 2 reveals the DFS and OS according to combined Ki-67 status; Pre-Ki-67 status may predict patient outcome even when discordant Ki-67 status existed in 23.9% of the cases.

## Discussion

The motivation behind this study was to investigate whether the timing of measuring Ki-67 levels affected the accuracy of evaluating tumor progression and aggression, along with predicting patient prognosis. In several previous studies, it has been argued that the Ki-67 levels ascertained from biopsies and surgical specimens are related. However, most of the papers compared and analyzed biopsies and surgical specimens of the same patient and demonstrated that both Ki-67 levels were concordant [16, 17, 22]. In contrast, there have been studies that suggested that changes between CNB and surgical specimen affect the prognosis of patients. However, our study did not include patients who had undergone neoadjuvant chemotherapy because the prognosis was confirmed through an independent group of changes in the overall Ki-67 level and not just a change in one patient’s Ki-67 level. Therefore, in this study, the Pre-Ki-67 (biopsy) and Post-Ki-67 (specimen) groups were independent without any overlap. We expected a proportional increase in the hazard ratio according to increased Ki-67 levels; however, since we confirmed that Ki-67 is heterogeneous, there was no proportionate increase in the hazard ratio in the Pre-Ki-67 or Post-Ki-67 groups. However statistically, the hazard ratio increased more significantly in the Post-Ki-67 group than in the Pre-Ki-67 group; thus, it can be inferred that Ki-67 from specimens is superior for prognostic evaluation. Despite these problems inevitably raised by the current design and nature of the study, independent groups showed similar prognostic patterns of Ki-67 levels, and it was proved that the Ki-67 level could predict the prognosis of breast cancer, regardless of the time of measurement. Moreover, this study showed that the Ki-67 index was related to different variables, such as larger tumor size, greater histologic grade, negative ER/PR status, and positive HER2 status [11, 12, 23, 24]. Therefore, the combination of Ki-67 and other variables should be further studied to validate our findings and develop a model that can predict prognosis more accurately.

Unlike previous prognostic studies, variables for therapeutic interventions were identified in the present study. In particular, with regard to local therapy along with proliferation, various other factors were considered. However, in the case of systemic therapy, the Ki-67 index tends to increase if a patient does not receive endocrine therapy and instead receives chemotherapy or biologic therapy. These results suggest that, regardless of the time of examination, the Ki-67 value indicates the final proliferative state of breast cancer and eventually indicates patient prognosis. Therefore, the treatment method can be modified depending on this result. A high-Ki-67 level reflects an advanced pathological state and shows that the subtype is more likely to be a basal-like or HER2-only type than a luminal type [10, 22, 25, 26].

The results of this study demonstrated that the higher the Ki-67 values, the higher the probability of receiving more aggressive treatment. After it was established that Ki-67 levels play a prognostic role in breast cancer, Cheang et al. proposed a cutoff value for Ki-67 as a prognostic factor and they evaluated this cutoff value for Ki-67 in several studies [7, 11, 12, 27, 28]. However, the methods of inspection and evaluation were insufficient due to variability between institutions, which precluded this cutoff from being included in a verified clinical guideline [9]. Recently, as inspection methods and evaluations have become automated, the objectivity of Ki-67 values has been secured to some extent; however, it is challenging to ensure standardization across all institutions [29, 30]. Our study proves that Ki-67 level is valuable as a prognostic factor despite such issues.

Many related papers have already evaluated the cutoff value of Ki-67 and this has also been discussed at the St. Gallen conference [11, 12, 15]. The reason why the sub-classification of the Ki-67 index in our analysis was conducted around 10%, 15%, and 20% is that in many studies related to Ki-67 cutoff values, the point for the cutoff is reported to be around 10–20% and we believed that significant changes in Ki-67 wound occur within that range [11, 12, 27, 28, 31]. In this study, a hazard ratio was not proportionate to the Ki-67 level. This is another reason why it is difficult to determine the cutoff value of Ki-67. Nevertheless, different prognostic management studies must be conducted considering a Ki-67 cutoff value of each institution in breast cancer patients under the same conditions in clinical practice to generate robust results. However, our main purpose is not to evaluate the prognosis through such a cutoff, but to show that, as an independent group, Ki-67 examined by Biopsy and surgical specimen has similar trends.

Our study had certain limitations related to the interpretation of results. The medium–low and medium–high groups had smaller sample sizes than that of the other Ki-67 subgroups; therefore, trend interpretation may be limited. Additionally, variables with similar properties to the Ki-67 level, particularly histologic grade, differed from the general results due to their overlapping with each other. As with other studies, a similar trend was observed in patients who underwent both tests and were similar to those in previous studies with a different prognosis according to the results of Ki-67 from biopsies rather than specimens. However, a limitation of this study is that accurate analysis is difficult due to the small sample size. Another inherent limitation was its single-center, retrospective study design.

Although many IHC biomarkers such as Ki-67 have been compared in biopsy and surgical specimens, multigene assays are also compared. Although it is not yet a study with the same concept as our study, there are studies that compare the results of the Oncotype DX from biopsy with from the surgical specimen and suggest that Oncotype DX from biopsy results is also concordant and reliable with the surgical specimen [32, 33]. As our future study, based on this study, we will begin prognostic study in luminal type and comparison of prognosis between Ki-67 and Oncotype DX or other multigene analysis.

However, to the best of our knowledge, this is one of the largest retrospective studies on the prognostic effectiveness of Ki-67; in addition to being a retrospective, non-metastatic breast cancer population without any prior selection bias. Additionally, performing pathological and biomarker evaluation in a single, well trained, and accredited laboratory was another advantage of this study, which demonstrated realistic values of IHC in clinical practice [16, 24]. Moreover, the fact that we considered variables related to treatment also differentiates the present study from previous ones.

## Conclusion

We found that the Ki-67 level is an important independent prognostic factor that is more accurate than any other factor, regardless of the timing of Ki-67 testing, i.e., tested preoperatively or postoperatively. In addition, even if there are methodological and analytical problems related to Ki-67 testing, evidently, there are no problems in clinical practice. And it is difficult to know which timing of Ki-67 testing is superior. However, considering the independent comparisons of groups that had only one test and groups that had both tests, it seems to be advantageous for breast cancer treatment to check the Ki-67 level preoperatively. In recent years, breast cancer treatment has become diverse, and early diagnosis and evaluation of disease are highly beneficial for recovery. Therefore, we recommend measuring the Ki-67 level in the preoperative period and measuring Pre-Ki-67 levels, which are currently measured with CNB and VAB specimens, as good clinical practice. However, it should be considered that the Ki-67 level observed in specimens may be slightly more accurate in reflecting the prognosis.

## Data Availability

The datasets generated and/or analyzed during the current study are available from the corresponding author on reasonable request.

## Abbreviations

BMI: Body mass index
CNB: Core needle biopsy
DFS: Disease-free survival
ER: Estrogen receptor-positive
FISH: Fluorescence in situ hybridization
IHC: Immunohistochemical
OS: Overall survival
PR: Progesterone receptor
SD: Standard deviation
SISH: Silver in situ hybridization
VAB: Vacuum-assisted biopsy
